# Pectin-modified PCN-222 for detecting nitrofurazone residues with dual signals in livestock, poultry, and aquatic products

**DOI:** 10.1016/j.fochx.2025.103253

**Published:** 2025-11-04

**Authors:** Keyu Du, Jie Shen, Siyao Zhong, Hefei Wang, Yulou Qiu, Xuping Shentu, Xiaoping Yu, Zihong Ye, Haizhi Huang

**Affiliations:** a Key Laboratory of Microbiological Metrology, Measurement & Bio-product Quality Security, State Administration for Market Regulation, College of Life Science, China Jiliang University, Hangzhou 310018, China; b Zhejiang Provincial Key Laboratory of Biometrology and Inspection and Quarantine, China Jiliang University, Hangzhou 310018, China

**Keywords:** Lateral flow immunoassay, Metal-organic frameworks, Nitrofurazone, Colorimetric-fluorescent immunoassay probe

## Abstract

Nitrofurazone and its metabolite semicarbazide (SEM) residues present in food products pose substantial health hazards to humans. Herein, the colorimetric–fluorescent lateral flow immunoassay (LFIA) for 4[(4-carboxyphenyl)-methylene]-hydrazinecarboxamide (CPSEM) detection was developed using PCN-222@Pectin as a signal tag. Herein, the PCN-222 surface was subsequently modified with pectin to enhance its aqueous solubility and stability. The PCN-222@Pectin-LFIA system exhibited a broad linear range from 0.025 ng/mL to 100 ng/mL. The low limits of detection for the colorimetric and fluorescence-based detection methods were 0.1 and 0.05 ng/mL, respectively. Notably, these values represent a 10- and 20-fold higher sensitivity than that of the traditional colloidal gold nanoparticle-based LFIA. This method was successfully used to detect CPSEM in actual samples, with recovery rates of 89.34 %–120.80 %. This approach fulfills the need for on-site preliminary qualitative assessment during detection and permits subsequent accurate quantitative analysis.

## Highlights

• A modified PCN-222 was synthesized for LFIA detection of nitrofurazone residues.
• The system offers a dual-mode colorimetric and fluorescent signal output.
• This method significantly enhances sensitivity compared to traditional GNPs-LFIA.
• This approach was implemented for CPSEM detection in animal derived food.

## Graphical abstract

**Figure:**
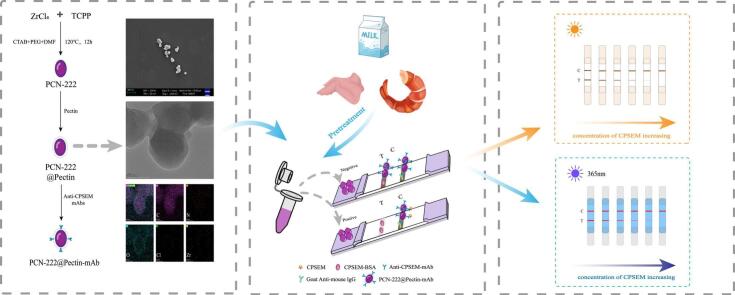

## 1 Introduction

Nitrofurans, a group of synthetic broad-spectrum antibiotics, include furaltadone(FTD), furazolidone(FZD), nitrofurantoin(NFT), and nitrofurazone(NFZ). Their efficacy arises from strong antibacterial properties against a wide range of Gram-positive and Gram-negative bacteria, along with select specific pathogens (Wu, Yang, Yu, & Li, 2020). These agents are widely applied in diverse sectors, including the livestock, aquaculture, and apiculture sectors (Miao et al., 2025; Wu, Yang, Liu, et al., 2020). NFZ has been used in livestock and aquaculture practices to manage multiple bacterial infections in animals (Thirumalraj et al., 2023). However, nitrofurans, represented by nitrofurazone, exhibit carcinogenic and teratogenic risks. Their toxic residues can accumulate in humans through dietary exposure pathways, and prolonged intake might result in adverse effects such as hemolytic anemia, polyneuritis, ocular damage, and acute liver necrosis (Suo et al., 2023). Consequently, numerous countries have enacted restrictive regulations governing such drug use. Since 1995, the European Union has banned the use of nitrofurans in livestock, poultry, and aquatic animals. The United States of America, within its federal regulations, has prohibited these drugs. The Ministry of Agriculture and Rural Affairs of the People's Republic of China released Announcement No. 193 in April 2002, banning nitrofurans in veterinary drugs. Metabolites are preferred over parent drugs as detection targets for nitrofuran antibiotics owing to their enhanced stability. These include 3-amino-5-morpholinomethyl-2-oxazolidinone (AMOZ), 3-amino-2-oxazolidinone (AOZ), semicarbazide (SEM), and 1-aminohydantoin (AHD). Announcement No. 250 of the Ministry of Agriculture and Rural Affairs of the People's Republic of China strictly prohibits detectable levels of these metabolites in food-producing animals. These metabolites are generated when the parent drug enters the organism and binds to tissue proteins during administration. Lacking further metabolic capacity, they accumulate in animal tissues (Jia et al., 2022). Thus, efficient and sensitive food testing methods for the detection of these substances in food are of great importance. Upon entering the biological system, nitrofurazone is promptly metabolized into SEM, which subsequently binds to proteins in the organism to form a highly stable complex, namely, 4[(4-carboxyphenyl)-methylene]-hydrazinecarboxamide (CPSEM). Therefore, CPSEM is adopted as the target for monitoring NFZ residues.

NFZ and its metabolites are detected using equipment-based and immunoassay methods. Equipment-based methods, particularly chromatographic methods, offer relatively high precision. However, the equipment is bulky and expensive, pretreatment operations are complex and require skilled operators, and detection costs are high (Cai et al., 2022). Immunoassay methods include Enzyme-Linked Immunosorbent Assay (ELISA), electrochemical immunoassay (ECIA), and lateral flow immunoassay (LFIA). ELISA is highly sensitive and specific, while ECIA combines high sensitivity and rapid response kinetics. LFIA represents a rapid paper-based detection platform using the principle of specific antigen–antibody interactions, exhibiting distinct advantages including rapid assay time, operational simplicity, and cost effectiveness. Typically, the assay can be completed within 10–20 min, facilitating its application in the detection of various antibiotics (Li, Wu, et al., 2021).

Gold nanoparticles (GNPs), a traditional probe carrier material for LFIA, have been used for the detection of multiple pharmaceutical residues. However, GNPs have limited precision, stability, quantification ability, and sensitivity (J. Wang et al., 2021). Advanced materials such as quantum dots, up-conversion, and metal nanoclusters have been successively explored and harnessed as substrate materials for probes within the LFIA. Notably, substantial sensitivity enhancement has been achieved (Aleem et al., 2024; Y. Wang et al., 2023; Zhang, Li, et al., 2023; Zhu et al., 2022). However, these materials face problems such as easy quenching, short fluorescence lifetime, and poor biocompatibility (Sobhanan et al., 2023). In LFIA, biocompatibility directly affects the coupling effect between the material and antibody, ultimately influencing the performance of the entire system. Therefore, it is crucial to seek new carrier materials. Metal-organic frameworks (MOFs), a new generation of porous materials assembled from metal nodes and organic linkers through interactions, are ideal candidates for sensing and detection. They are favored owing to their distinct electro-optical attributes, large pore sizes, high specific surface areas, excellent biocompatibility, and ease of modification (Lazaro & Forgan, 2019). Notably, through straightforward ligand modification strategies, diverse fluorescent materials can be purposefully introduced into MOF nanopores, endowing them with unique fluorescent properties (Han et al., 2024; Mohanty et al., 2024). The distinct porous architecture of MOFs alleviates challenges in antibody conjugation, enhancing the synthetic efficiency of immunoprobes. PCN-222, also known as MOF-545 or MMPF-6, is a zirconium (Zr)-based MOF comprising Zr nodes and tetrakis(4-carboxyphenyl)porphyrin (TCPP) ligands. Among numerous MOF materials, PCN-222 exhibits the largest open channels and demonstrates exceptional chemical stability, retaining structural integrity even after 24 h storage in concentrated hydrochloric acid solution(HCl) (Zhang et al., 2023). Owing to the presence of TCPP, PCN-222 appears purple under visible light and emits a vivid red fluorescence when exposed to 365-nm ultraviolet (UV) light, making it ideal for the development of colorimetric–fluorescent bimodal biosensors. Current research has used PCN-222 for the detection of phosphates and FZD metabolites (Cheng et al., 2020; Zhong et al., 2024). Nevertheless, its poor hydrophilicity and potential porphyrin leakage limit the development of a LFIA platform (J. Chen et al., 2021). Therefore, to address these drawbacks, we attempted to coat its surface with a pectin film, where pectin is a natural, biocompatible polysaccharide endowed with intrinsic biological activity. It features a complex structural framework comprising well-defined domains such as rhamnogalacturonan-I, homogalacturonan, rhamnogalacturonan-II, and xylogalacturonan (Polesca Freitas et al., 2021). The pectin molecular chain contains a large number of functional hydroxyl and carboxylic acid groups, which significantly enhance the pectin hydrophilicity and the potential for modifying other molecules. Thus, it has become indispensable in biomedical applications and pharmaceutical delivery (Li, Li, et al., 2021; Rajabzadeh-Khosroshahi et al., 2025). Herein, pectin was coated on the surface of PCN-222 to prepare a composite material, namely, PCN-222@Pectin. A colorimetric–fluorescent dual-output LFIA system was established for the detection of the NFZ metabolite derivative CPSEM in food. This system was effectively applied for the detection of CPSEM in milk, chicken, and shrimp samples.

## 2 Experiment

### 2.1 Materials and reagents

Zirconium chloride (ZrCl_4_)(≥99.9 %), benzoic acid (BA)(≥99.5 %), TCPP(≥97 %), *N*,*N*-dimethylformamide (DMF)(≥99.9 %), absolute ethanol(≥99.8 %), polyethylene glycol 6000 (PEG 6000), hexadecyl trimethyl ammonium bromide (CTAB)(≥99 %), dimethyl sulfoxide (DMSO)(≥99.9 %), pectin, and bovine serum albumin (BSA)(≥98 %) were obtained from Aladdin Chemistry Co., Ltd. (Shanghai, China). Antibodies to CPSEM were purchased from Wuxi Ditengmin Biotechnology Co. Anti-mAb was purchased from Hebei Lanfei Biotechnology Co. Sample pads, nitrocellulose (NC) membranes, polyvinyl chloride (PVC) plates and absorbent pads were purchased from Shanghai Gold Standard Biotechnology Co. in Shanghai, China. SEM, CPSEM, clenbuterol hydrochloride, and oxytetracycline (OXY) that meet analytical standards were purchased from Anpel Standard Technical Service Co., Ltd. (Shanghai, China). Milk, chicken and shrimp were purchased from local supermarkets (Hangzhou, Zhejiang).

BRUKER OPUS-7.5 Fourier transform infrared spectroscopy (Bruker GMBH, Germany), sigma300 field emission scanning electron microscope (Zeiss, Germany), Q/SGYM 1009 electronic balance (Ohaus Instrument Co., LTD.), KQ-250B type ultrasonic cleaner (Kunshan Ultrasonic Instrument Co., LTD.), TG16G Kaida centrifuge (Hunan Kaida Scientific Instrument Co., LTD.), XW-80 A vortex mixer (Shanghai Jingke Industrial Co., LTD.), VELOCITY 18R pro desktop multi-functional centrifuge (Techcomp, Britain) and ZF-90B multifunctional UV projector (Shanghai Guanghao Analytical Instrument Co., LTD); Talos F200S (FEI US); X-ray diffractometer D8 ADVANCE (Bruker, Germany); Zetasizer Nano ZS90 (Malvern, Britain); all LFIA grayscale values are read using Image J software.

### 2.2 Synthesis of nanomaterials

PCN-222 was synthesized in accordance with previous studies with slight modifications (Liu et al., 2017). BA (200 mg), ZrCl₄ (20 mg), CTAB (66.7 mg), TCPP (6.67 mg) and PEG (66.7 mg) were dissolved in DMF (6.67 mL) under ultrasonic conditions for 10 min. The mixed solution was transferred to a 25-mL Teflon-lined autoclave and heated at 120 °C for 12 h. After cooling to room temperature, the mixture was centrifuged at 20970*g* for 30 min at 4 °C. The supernatant was discarded, and the precipitate was washed three times with DMF, absolute ethanol, and ultrapure water, respectively. Finally, the resulting product was dissolved in ultrapure water and stored in the dark.

PCN-222@Pectin was prepared following reported protocols with some modifications (Tang et al., 2019). 125 mg of pectin powder was dissolved in 500 mL of ultrapure water and stirred at 500 rpm for 0.5 h at 70 °C to ensure the complete dissolution. After cooling to room temperature, the pH of the pectin solution was adjusted to 6, and the solution was filtered through a 0.8-μm membrane. The prepared PCN-222 (2.5 mL) was diluted to 25 mL with ultrapure water and subjected to ultrasonic treatment for 1 h. This solution was then added dropwise to the pectin solution, followed by overnight stirring at 500 rpm. The following day, the mixed solution was centrifuged at 20970 *g* for 30 min at 4 °C, and the precipitate was washed three times with ultrapure water. Finally, the product was redissolved in ultrapure water to obtain PCN-222@Pectin.

The colloidal gold solution was synthesized via the trisodium citrate reduction method (Fang et al., 2023). Initially, 100 mL of a 0.01 % (*w*/*v*) tetrachloroaurate(III) (HAuCl_4_) was added to a 250-mL round-bottom flask and heated to boiling under reflux using a heating mantle, with constant magnetic stirring. Subsequently, 2.25 mL of a 1 % (w/v) trisodium citrate solution was rapidly injected into the boiling solution under vigorous stirring. The reaction system was maintained under reflux with continuous stirring for 15 min to ensure complete reduction of chloroaurate ions. After cooling to room temperature, the resultant wine-red colloidal gold solution was aliquoted into sterile centrifuge tubes and stored at 4 °C for subsequent experiments.

### 2.3 Preparation of Immunoprobes

PCN-222@Pectin-mAb probe was fabricated following a reported method (Tian et al., 2021). A suitable amount of PCN-222@Pectin was diluted with ultrapure water and sonicated for 10 min to obtain a homogeneous solution. Then, 10 μg of antiCPSEM-mAbs was added to 1 mL of PCN-222@Pectin solution (0.072 mg/mL) and the mixture was incubated with gentle shaking at room temperature for 40 min. Next, 100 μL of 10 % BSA solution was incorporated to block unbound sites and the mixture was shaken at room temperature for 30 min. Later, the mixture was transferred to a 4 °C refrigerator for 2 h of incubation. Besides, it was centrifuged at 19350 *g* at 4 °C for 15 min to remove the unbound proteins. Finally, the supernatant was discarded, and the obtained precipitate was resuspended in the phosphate-buffered saline (PBS) buffer (100 μL) and stored in a refrigerator.

The GNPs-mAb probe was synthesized using a previously reported method with some modifications (C. Chen et al., 2024). Specifically, 1 mL of the colloidal gold solution was aliquoted and adjusted to the optimal pH using 0.2-M potassium carbonate (K_2_CO_3_). Then, 6 μL of CPSEM-mAbs (1 mg/mL) was added to the activated colloidal gold suspension, followed by 40-min incubation at room temperature (25 °C) with gentle agitation. To block residual reactive sites, 100 μL of a 10 % (*w*/*v*) BSA was added, and the mixture was further incubated for 30 min under identical conditions. The conjugated nanoparticles were then isolated by centrifugation at 12000 *g* for 30 min at 4 °C. The resulting pellet was carefully resuspended in PBS and stored at 4 °C in the dark until use.

### 2.4 Construction of the Immunochromatographic system

Immunochromatographic test strips are typically constructed from a sample pad, nitrocellulose membrane, absorbent pad, and a backing plate. The sample pad was presoaked in a 2 % BSA solution and dried overnight at 37 °C. CPSEM-BSA and goat antimouse IgG (1 mg/mL) were dispensed onto the NC membrane as the T-line and C-line, respectively, at a rate of 0.8 μL/cm. The membrane was subsequently dried at 37 °C for 1 h. The components were assembled, cut into 4 mm-wide strips and stored at 4 °C in the dark.

### 2.5 Performance evaluation of PCN-222@pectin-LFIA

For performance evaluation, 100 μL of the diluted CPSEM standard substances (with concentrations of 0.025, 0.05, 0.1, 0.25, 0.5, 1.0, 2.5, 5.0, 10, 25, 50, 100 ng/mL) and deionized water were mixed with the PCN-222@Pectin-mAb probe. The test strip's sample pad was submerged in the solution for 15 min, after which brownish-yellow bands were visually assessed for qualitative results while red fluorescent bands were observed under 365 nm UV illumination. Strip images were captured using a smartphone, followed by quantitative signal analysis with ImageJ software and fitting using a four-parameter logarithmic equation (Bai et al., 2022):$y=A_{2}+\frac{A_{1}-A_{2}}{1+{\left(\frac{x}{x_{0}}\right)}^{p.}}$

where *A*_*1*_ denotes the asymptotic mini_m_um value, *A*_*2*_ denotes the asymptotic maximum value, *p* represents the slope of the curve, and *x*_*0*_ corresponds to the concentration of the analyte at the inflection point.

Commonly used antibiotics in the livestock industry such as CPAHD, CPAOZ, CPAMOZ, clenbuterol hydrochloride, and oxytetracycline were selected. All of these antibiotics were diluted to 100 ng/mL. The specificity of PCN-222@Pectin-LFIA was evaluated through cross-experiments.

### 2.6 Sample pretreatment and analysis

In consideration of the reliability of the experiment, three typical representatives (milk, chicken, and shrimp) were selected as real samples for detection. The treatment methods for these samples were referred to previous literature with some modifications (Zheng et al., 2024): 2 mL of skim milk was diluted 5-fold and then sonicated for 10 min. Then, 200 μL of 0.05 mol/L 4-carboxybenzaldehyde (4-CBA) in DMSO was added, and the mixture was incubated at 37 °C for 16 h. After centrifugation at 15533 *g* for 10 min, the supernatant was collected. For chicken and shrimp analysis, 0.5 g of blended tissue was loaded into the tube, followed by sequential addition of 2 mL SEM standards, 0.5 mL HCl, and 100 μL 4-CBA. The homogenate was incubated at 40 °C for 2 h, then cooled to room temperature and mixed with K₂HPO₄ and ethyl acetate. After vortexing thoroughly and centrifuging at 6213 g for 10 min at 4 °C, the ethyl acetate layer was evaporated to dryness. The residue was resuspended in n-hexane and PBST, vortexed, and centrifuged again. Finally, 60 μL of the lower layer was collected for subsequent testing.

### 2.7 Processing of data

The schematic diagrams of the experiment were generated using Maxon Cinema 4D 2023 and Adobe Illustrator 2023. The experimental data were processed, and the graphs were plotted using Microsoft Excel 2010 and Origin 2025b.

## 3 Results and discussion

### 3.1 Characterization of MOF materials

**Fig. 1:**
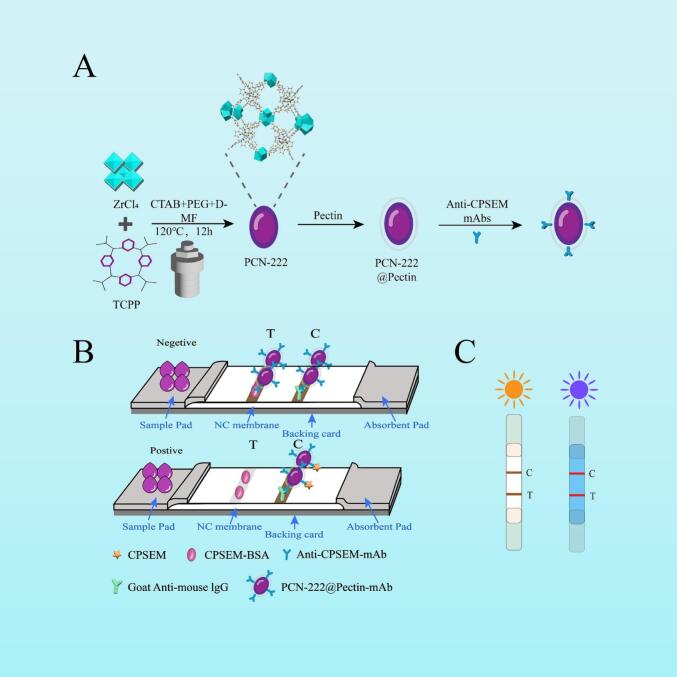
The synthetic procedure of PCN-222@Pectin-LFIA. (A) Schematic illustration of the synthesis steps of PCN-222, PCN-222@Pectin, PCN-222@Pectin-mAb; (B) Working principle of the PCN-222@Pectin-mAb-LFIA for detecting CPSEM; (C) Schematic illustration of the Immunostrips under Visible Light and under a 365-nm Ultraviolet Lamp.

**Fig. 2:**
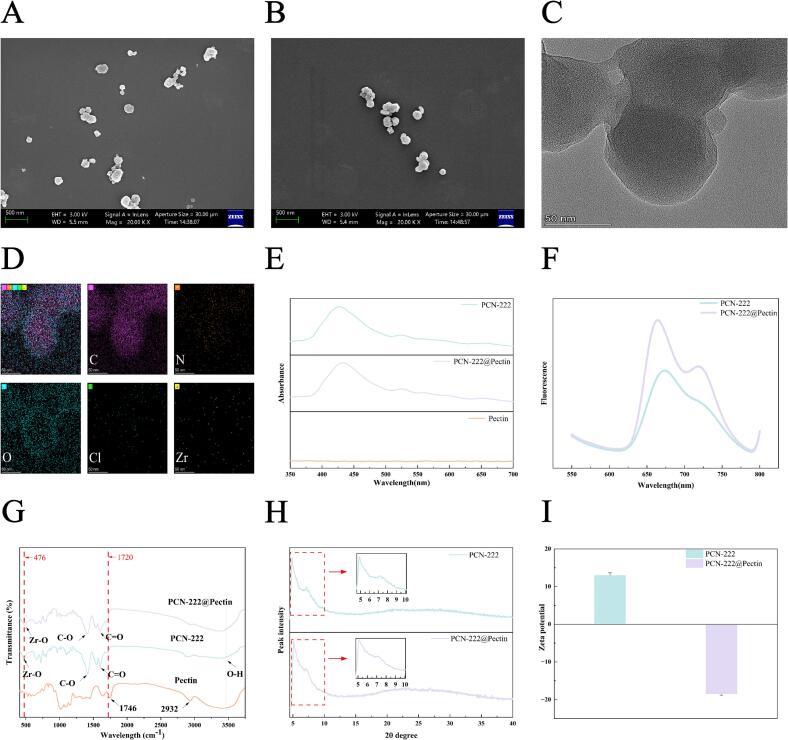
Characterization of MOF Materials. scanning electron microscope images of (A) PCN-222; (B) PCN-222@Pectin; (C) transmission electron microscope images of PCN-222@Pectin; (D) elemental mappings of C, N, O, Cl, Zr of PCN-222@Pectin; (E) UV–vis absorption spectra of PCN-222, PCN-222@Pectin and Pectin; (F) Fluorescence spectra of PCN-222 and PCN-222@Pectin; (G) FT-IR spectra of PCN-222, PCN-222@Pectin and Pectin; (H) XRD spectra of PCN-222 and PCN-222@Pectin; (I) Zeta potential of PCN-222 and PCN-222@Pectin.

The synthetic procedure of PCN-222@Pectin-mAb is depicted in Fig. 1A. Initially, PCN-222 was fabricated under high-temperature conditions. A pectin film was coated on the outer surface, yielding PCN-222@Pectin. Finally, mAbs were adsorbed onto the surface of the material using the shaking incubation process for 40 min to form PCN-222@Pectin-mAb. The scanning electron microscopy and transmission electron microscopy images presented in Figs. 2A and S1 reveal that the shape of PCN-222 is intermediate between spherical and oval, consistent with the reported results (Liu et al., 2017). After coating the outer layer with pectin, the surface of PCN-222@Pectin becomes smoother with the particle size of 100–200 nm (Fig. 2B and C) and no significant structural changes. Fig. 2D illustrates the distribution of carbon (C), nitrogen (N), oxygen (O), chlorine (Cl), and zirconium (Zr) in PCN-222@Pectin. The spectral properties of the materials, particularly the UV absorption and fluorescence spectra, were examined systematically. The UV absorption spectra (Fig. 2E) reveal that PCN-222 and PCN-222@Pectin exhibit a maximum absorption peak at 420 nm owing to the presence of TCPP. Furthermore, four distinct characteristic shoulder peaks are observed in the range of 500–700 nm. The fluorescence spectrum, obtained at an excitation wavelength of 420 nm, is depicted in Fig. 2F, featuring a prominent emission peak at 675 nm.

The Fourier-transform infrared spectra (Fig. 2G) indicate that the peak at 476 cm^−1^ represents the Zr—O bond, while the peaks located at 1420 cm^−1^ and 1600 cm^−1^ correspond to the C—O bond and C=O bond of the carboxyl group, respectively. The peak at 1720 cm^−1^ disappears after pectin coating indicating the successful encapsulation of PCN-222 within pectin.

Powder X-ray diffraction (PXRD) was used to analyze the crystalline properties of the synthesized materials (Fig. 2H). The characteristic PXRD diffraction patterns of PCN-222 and PCN-222@Pectin exhibited distinct characteristic peaks, which match with the reported results (Liu et al., 2017), confirming the materials' successful synthesis.

Pectin-coated PCN-222 exhibits a transition of the zeta potential from positive to negative values (Fig. 2I). Notably, an increase in the absolute value of the zeta potential indicates the successful synthesis of PCN-222@Pectin and corroborates that the dispersibility of PCN-222@Pectin in an aqueous medium is more favorable than that of PCN-222.

### 3.2 Optimization of LFIA parameters

**Fig. 3:**
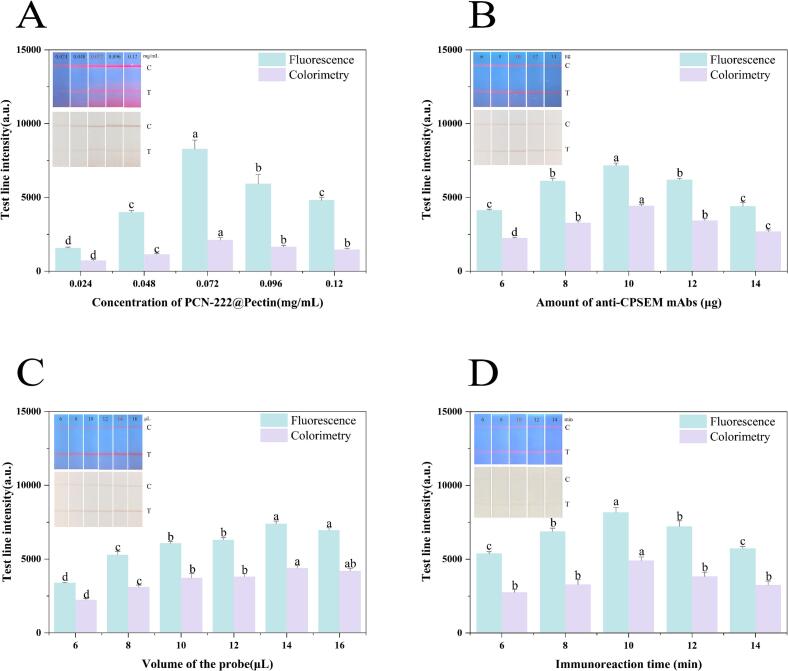
Optimization of PCN-222@Pectin-LFIA parameters. The effect of (A) the concentration of PCN-222@Pectin; (B) the amount of anti-CPSEM mAbs; (C) the volume of the PCN-222@Pectin-mAb probe; (D) the immunoreaction time for the detection of CPSEM, the same line with different letters represented significant differences (*P* < 0.05).

To enhance the detection performance of the immunochromatographic test strip, several key parameters were optimized. Herein, the colorimetric-fluorescence intensity method of the test line (T-line) was used to assess the optimal parameters (Fig. 3), and the control line (C-line) was used to verify the validity of the results.

PCN-222@Pectin serves as the source of colorimetric and fluorescent signals, and its concentration strongly impacts detection results. Low concentrations will result in a weak signal, while high concentrations may reduce the efficiency of its coupling with antibodies. We first optimized the PCN-222@Pectin concentration. Fig. 3A shows that the strongest T-lines for fluorescence and colorimetry are optimal at 0.072 mg/mL, following which they are weakened.

During the probe preparation process, the amount of antibody may influence the capture efficiency of the conjugates. To achieve satisfactory detection performance, the optimal coupling amount of antiCPSEM-mAbs was investigated over the range of 6–14 μg per probe. Fig. 3B shows that when the amount of antiCPSEM-mAbs is 10 μg, the colorimetric and fluorescence intensity of the T-line reaches its peak. Therefore, 10 μg was selected as the optimal dosage of the antibody.

The probe volume directly affects the experimental results. Within a defined range, increasing it helps the target antigen to capture the probe effectively, increasing detection sensitivity. However, excessive probe concentrations might cause a high background noise and related problems. The volume of the probe was systematically optimized within the range of 6–16 μL. Fig. 3C shows that as the probe volume increases, the signal output intensities for colorimetric and fluorescent assays continuously increase. When the probe volume reaches 14 μL, the signal output intensity of the T-line attains its maximum value. Consequently, 14 μL was designated as the optimal volume for PCN-222@Pectin-mAb.

The immunoreaction duration is crucial for antigen-antibody complex formation. Both excessively long and short durations can adversely affect assay performance. Specifically, a short duration may result in insufficient antigen-antibody binding, leading to a weak signal. Conversely, an extended immunoreaction duration may cause the elution effect to occur. Upon the initiation of the reaction, the colorimetric and fluorescent intensities of the T-line were captured and documented at 6, 8, 10, 12, and 14 min, respectively. Fig. 3D shows that at a reaction time of 10 min, the intensities of T-lines for colorimetry and fluorescence reach their peak values. Therefore, 10 min was established as the optimal reaction time for the PCN-222@Pectin-LFIA.

### 3.3 Sensitivity determination and specificity analysis

Sensitivity and specificity are crucial parameters for evaluating the PCN-222@Pectin-LFIA. As it generates both colorimetric and fluorescent dual-output signals, the colorimetric output signal was used for preliminary qualitative research, whereas the intensity of the fluorescent signal served as the basis for precise quantification.

**Fig. 4:**
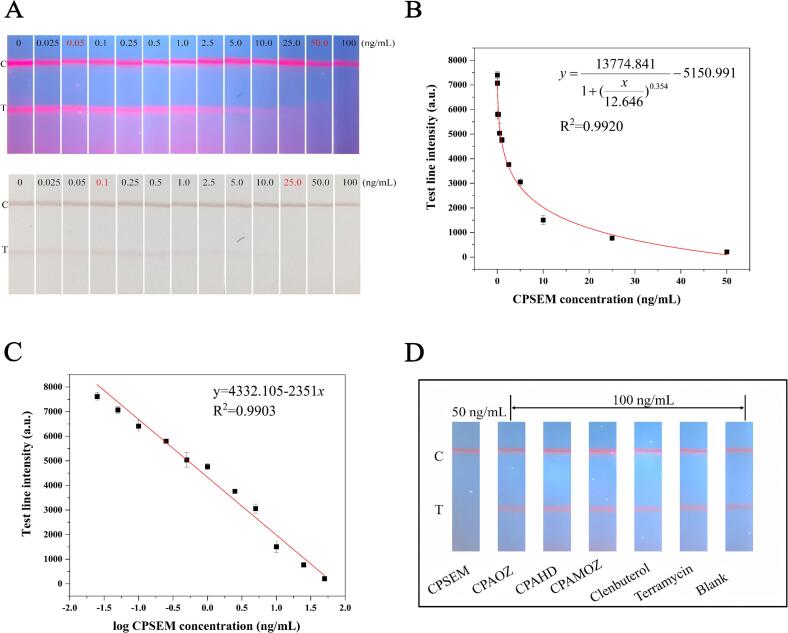
Sensitivity Determination and Specificity Analysis of PCN-222@Pectin-LFIA. (A) Sensitivity results of PCN-222@Pectin-LFIA for CPSEM detection; (B) Calibration curve of CPSEM detection with a concentration ranging from 0.025 to 100 ng/mL; (C) The variation rule of CPSEM in the range of 0–100 ng/mL using the PCN-222@Pectin-LFIA; (D) Specificity of the PCN-222@Pectin-LFIA for detecting CPSEM, 50 ng/mL of CPAOZ and 100 ng/mL of other antibiotics.

The visual limit of detection (vLOD) was defined as the lowest CPSEM concentration that causes a significant reduction in T-line fluorescence when compared to that of negative controls. The cut-off value was defined as the concentration at which T-line fluorescence vanishes entirely (Tian et al., 2021). To investigate the sensitivity, a series of CPSEM standard solution groups with varying concentrations (0, 0.025, 0.05, 0.1, 0.25, 0.5, 1, 2.5, 5, 10, 25, 50, and 100 ng/mL) was established. Fig. 4A illustrates that the fluorescence intensity of the T-line decreases correspondingly with the continuous increase in the concentration of CPSEM. At a CPSEM concentration of 0.05 ng/mL, the fluorescence intensity of its T-line is significantly weaker than that of the negative test strip. When the concentration reaches 50 ng/mL, the fluorescence on the T-line vanishes entirely. Consequently, the vLOD and cut-off values for PCN-222@Pectin-LFIA are 0.05 and 50 ng/mL, respectively. Furthermore, a typical calibration curve was derived based on the four-parameter logarithmic equation of PCN-222@Pectin-LFIA mentioned previously, exhibiting a relative coefficient R^2^ of 0.992 (Fig. 4B). A significant linear dependence exists between the T-line fluorescence intensity and logarithmic CPSEM concentration across the range of 0.025–100 ng/mL, with the R^2^ of 0.9903 (Fig. 4C).

Specificity is another crucial indicator for validating the LFIA, as it can evaluate the method's ability to resist interference. Fig. 4D shows five common drugs used as potential interferents for the specificity assessment. The results indicate that the presence of the target analyte eliminates the T-line on the test strip. In contrast, when interferents are present, the T-line on the strip exhibits a strong signal. These results reveal that PCN-222@Pectin-mAb can completely differentiate the target from its structurally similar analogs, thereby demonstrating excellent specificity of the monoclonal antibody.

### 3.4 Stability and repeatability of the method

The stability of PCN-222@Pectin was analyzed under variable temperature, storage, and pH conditions (Fig. S2). The performance of this material did not show any significant changes within the temperature range used herein (4 °C–80 °C, Fig. S2A). Fig. S2B shows that the material can be stored at room temperature for an extended period without significant property deterioration. Stability evaluation of PCN-222@Pectin under varying pH conditions was further conducted (Fig. S2C). The results demonstrate that PCN-222@Pectin exhibits structural and fluorescent stability over a pH range of 5.0–8.0. When pH is less than 5.0, strongly acidic conditions induce probe inactivation, whereas alkaline environments at pH > 8.0 structurally degrade the material and TCPP leakage.

Fig. S3 depicts the intraassay and intraday repeatability results of the method, confirming its excellent repeatability.

### 3.5 Application in real samples

**Fig. 5:**
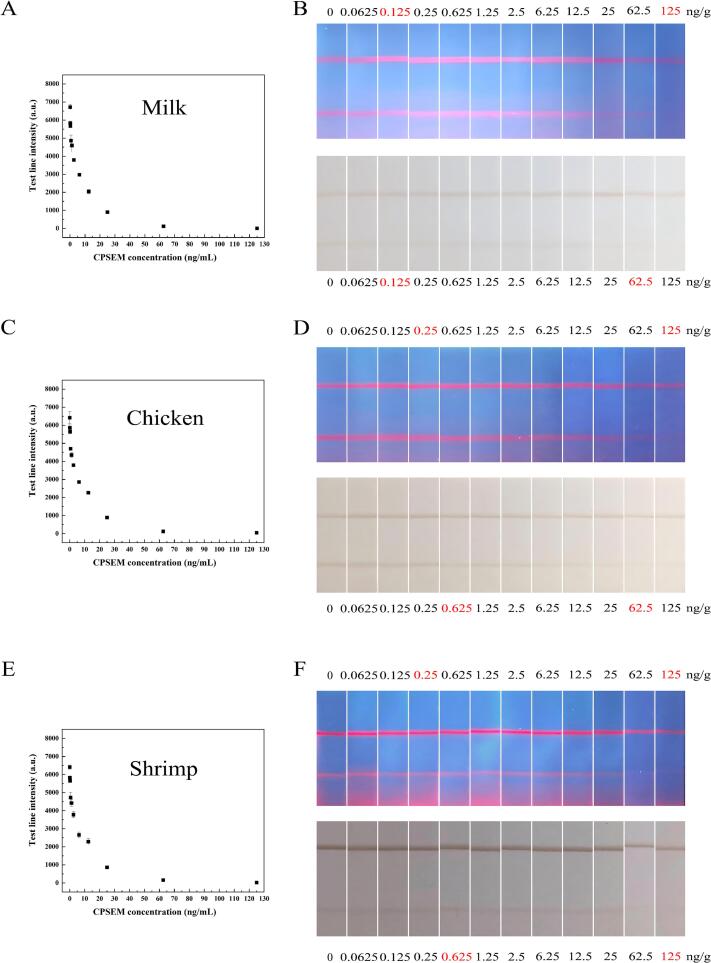
Application of PCN-222@Pectin-LFIA in real samples. Application of PCN-222@Pectin-LFIA to actual samples (fluorescent detection). (A-B) milk, (C—D) chicken and (*E*-F) shrimp samples.

Milk, chicken, and shrimp were selected to evaluate the practical application of this method. SEM standards at varying concentrations were spiked into these three samples, followed by derivatization operations. The average recoveries and relative standard deviations (RSDs) of CPSEM are presented in Table 1. For the three types of samples, the average recoveries of CPSEM from spiked samples range from 89.34 % to 120.80 %, with all RSDs of <13.0 % (Fig. 5). These experimental results demonstrate that the proposed method exhibits good accuracy and reliability for the detection of CPSEM in these spiked samples.

**Table 1:** Spiking results of CPSEM detected by PCN-222@Pectin-LFIA in food samples (*n* = 3).

Sample	Added (ng/g)	Mean found±SD (ng/g)	Recovery (%) ± RSD (%)
Milk	0.25	0.29 ± 0.01	117.47 ± 3.64
	2.5	2.45 ± 0.02	98.08 ± 0.88
	25	21.59 ± 0.60	89.34 ± 0.53
Chicken	0.25	0.30 ± 0.04	120.80 ± 11.84
	2.5	2.46 ± 0.06	98.50 ± 2.45
	25	22.67 ± 0.45	90.7 ± 1.99
Shrimp	0.25	0.30 ± 0.03	119.5 ± 8.38
	2.5	2.58 ± 0.34	103.3 ± 13.0
	25	23.1 ± 0.07	92.6 ± 0.31

### 3.6 Comparison with other methods

The PCN-222@Pectin-LFIA method, developed for the detection of CPSEM in food, was compared with methods reported in previous literature. Furthermore, GNPs-LFIA was used for comparison purposes. All results are presented in Table 2, Figs. S4 and S5. In comparison with large-scale instrumental detection methods, the established PCN-222@Pectin-LFIA meets the sensitive detection requirements and features simpler operation, lower cost, and shorter detection time. Compared with other immunochromatographic methods, the PCN-222@Pectin-LFIA established in this experiment exhibits two output modes (colorimetric and fluorescent), along with high sensitivity (vLOD = 0.05 ng/mL) and a wide analysis range (0.025–100 ng/mL). Additionally, in the analysis of actual samples, this method also demonstrates excellent performance. Therefore, PCN-222@Pectin-LFIA retains the advantages of traditional lateral flow immunochromatography, such as low cost, simple operation, and sensitive detection, and improves the sensitivity and quantification ability for detecting trace antibiotic residues.

**Table 2:** Method comparison with CPSEM detection.

Method	Test substance	vLOD or LOD	Liner range	Reference
UHPLC-FLD	SEM	0.22 ng/g	1–40 ng/g	(K. Wang et al., 2022)
UPLC-MS/MS	NPSEM	0.058 ng/g	0.02–5 ng/g	(Regan et al., 2022)
UHPLC-QTOF- MS/MS	NPSEM	0.27 ng/g	1–50 ng/g	(Dong et al., 2023)
QBs-RFLFIA	NPSEM	0.5 ng/mL	0–10 ng/mL	(Y. Cheng et al., 2023)
Au@4-MBN@AgNRs -LFIA	NPSEM	0.02 ng/g	0–1 ng/g	(Yanli Tian et al., 2024)
EuNPs-LFIA	NPSEM	0.1 ng/g	0–2.5 ng/g	(X. Dong et al., 2019)
SDA-based-LFIA	CPSEM	7.20 ng/L	0–10 ng/L	(W. Wu et al., 2019)
GNPs-LFIA	CPSEM	1 ng/mL	0.25–500 ng/L	This work
PCN-222@Pectin-LFIA	CPSEM	0.05 ng/mL	0.025–100 ng/mL	This work

However, the present investigation exhibits certain limitations, primarily encompassing operational complexity and prolonged processing duration inherent to the derivatization step within the pretreatment protocol. Accordingly, subsequent studies will focus on integrating advanced methodologies such as microwave-assisted technology to accelerate the derivatization process, while concurrently developing specialized instrumentation for the detection phase to optimize analytical workflow efficiency.

## 4 Conclusion

Herein, we report on the development of a simple, sensitive and dual-mode PCN-222@Pectin-LFIA for the detection of CPSEM in food matrices. By coating PCN-222 with a pectin film, the performance of PCN-222 was enhanced, resulting in a colorimetric-fluorescent probe suitable for LFIA. Preliminary qualitative detection was conducted under visible light, while accurate quantitative analysis was conducted by measuring the fluorescence signal intensity under 365-nm UV light irradiation. The method achieved vLOD values of 0.1 and 0.05 ng/mL for colorimetric and fluorescent modes, respectively. Additionally, it was successfully applied for the detection of CPSEM in milk, chicken, and shrimp samples. Future research will focus on shortening the overall processing time by reducing the derivatization step duration. In summary, the proposed PCN-222@Pectin-LFIA provides an effective method for detecting CPSEM and holds potential for monitoring other food and environmental pollutants.

## CRediT authorship contribution statement

**Keyu Du:** Writing – original draft, Methodology, Formal analysis, Conceptualization. **Jie Shen:** Methodology, Investigation. **Siyao Zhong:** Methodology, Investigation, Formal analysis. **Hefei Wang:** Methodology, Formal analysis. **Yulou Qiu:** Methodology, Investigation. **Xuping Shentu:** Validation, Supervision, Resources, Investigation. **Xiaoping Yu:** Visualization, Data curation. **Zihong Ye:** Validation, Supervision. **Haizhi Huang:** Validation, Supervision, Resources, Investigation.

## Declaration of competing interest

The authors declare that they have no known competing financial interests or personal relationships that could have appeared to influence the work reported in this paper.

## Data Availability

Data will be made available on request.

## Appendix A Supplementary data

Supplementary material Image 1
