# Supplementary material for: Pectin-modified PCN-222 for detecting nitrofurazone residues with dual signals in livestock, poultry, and aquatic products
Source: Food Chem X. 2025 Nov 4;32:103253. doi: 10.1016/j.fochx.2025.103253 (PMC12766102; doi:10.1016/j.fochx.2025.103253)
Supplement: Supplementary file 1 — Supplementary material [file mmc1.docx]

**Supporting Information for:**

**Pectin-modified PCN-222 for the detecting nitrofurazone residues with dual signals in livestock, poultry, and aquatic products**

Keyu Du^a,b^, Jie Shen^a,b^, Siyao Zhong^a,b^, Hefei Wang^a,b^, Yulou Qiu^a,b^, Xuping Shentu^a,b^, Xiaoping Yu^a,b^, Zihong Ye^a,b^, Haizhi Huang^*,a,b^

^a^Key Laboratory of Microbiological Metrology, Measurement & Bio-product Quality Security, State Administration for Market Regulation, College of Life Science, China Jiliang University, Hangzhou, 310018, China

^b^Zhejiang Provincial Key Laboratory of Biometrology and Inspection and Quarantine, China Jiliang University, Hangzhou, 310018, China

*Correspondence to:

Dr. Haizhi Huang

E-mail: [huanghz@cjlu.edu.cn](mailto:huanghz@cjlu.edu.cn).

**

**

Fig. S1. (A) TEM images of PCN-222; (B) elemental mappings of C, N, O, Cl, Zr of PCN-222





Fig. S2. The stability of (A) the PCN-222@Pectin under various temperature conditions, (B) the PCN-222@Pectin after being stored for different times, (C) the PCN-222@Pectin at different pH.





Fig. S3. The repeatability of PCN-222@Pectin-LFIA. (A) Intraassay repeatability; (B) Intraday repeatability


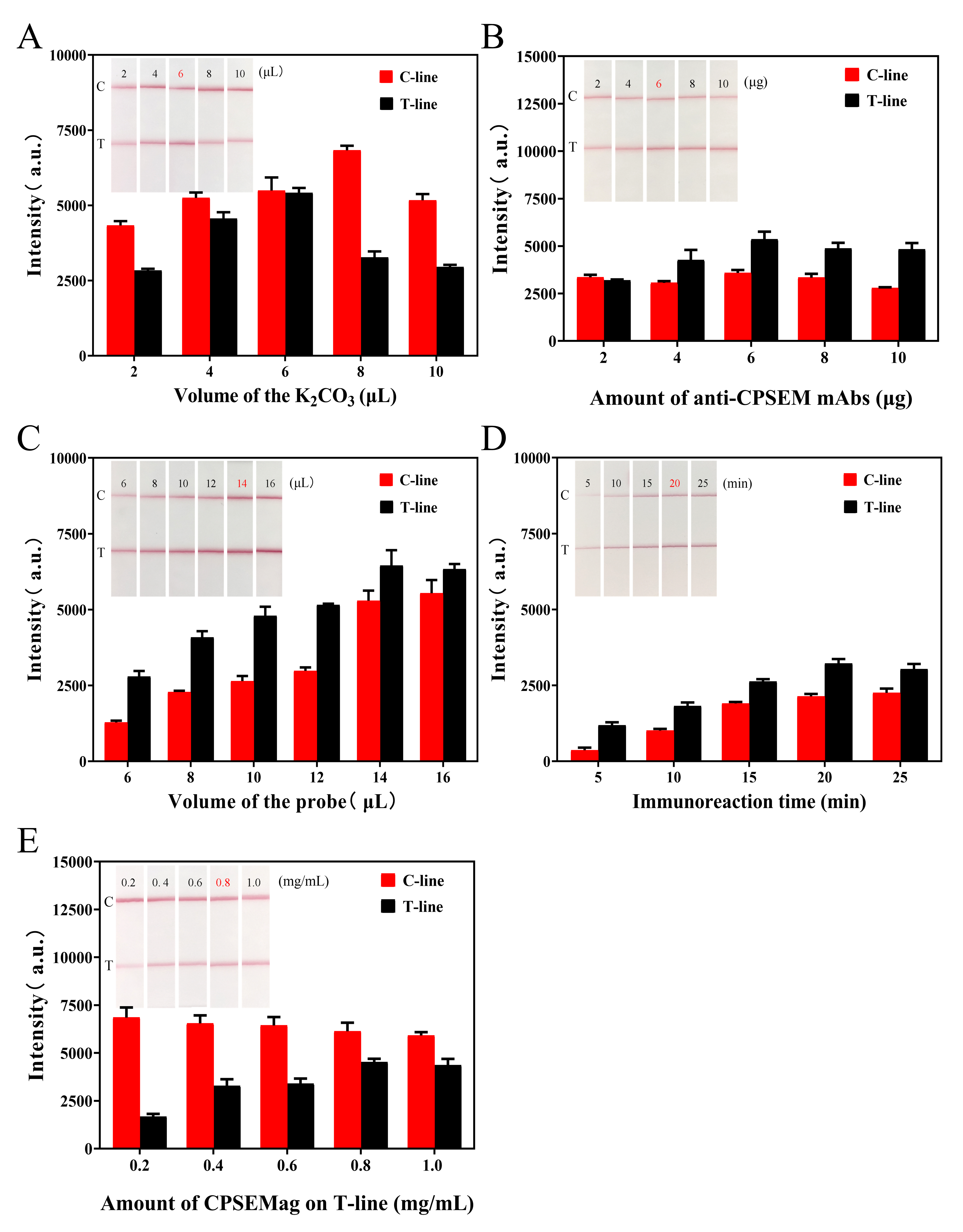


Fig. S4. Optimization of the GNPs-LFIA experimental parameters. The effect of (A) the volume of the K_2_CO_3_; (B) the amount of anti-CPSEM mAbs; (C) the volume of the GNPs-mAb probe; (D) the immunoreaction time; (E) the amount of CPSEM antigen on T-line for the detection of CPSEM.

**

**

Fig. S5. Sensitivity determination and specificity analysis of GNPs-LFIA experimental parameters. (A) Sensitivity results of GNPs-LFIA for CPSEM detection; (B) The variation rule of CPSEM in the range of 0-500 ng/mL using the GNPs-LFIA; (C) Calibration curve of CPSEM detection with a concentration ranging from 0.25-500 ng/mL; (D) Specificity of the GNPs-LFIA for detecting CPSEM, 0.5 μg/mL of CPSEM and 1 μg/mL of other antibiotics.
